# Roles of gap junctions, connexins, and pannexins in epilepsy

**DOI:** 10.3389/fphys.2014.00172

**Published:** 2014-05-07

**Authors:** Shanthini Mylvaganam, Meera Ramani, Michal Krawczyk, Peter L. Carlen

**Affiliations:** Neurobiology, Toronto Western Research Institute, University Health Network and University of TorontoToronto, ON, Canada

**Keywords:** gap junctions, connexins, pannexins, epilepsy, neurons, glia, animal models, human cerebral tissue

## Abstract

Enhanced gap junctional communication (GJC) between neurons is considered a major factor underlying the neuronal synchrony driving seizure activity. In addition, the hippocampal sharp wave ripple complexes, associated with learning and seizures, are diminished by GJC blocking agents. Although gap junctional blocking drugs inhibit experimental seizures, they all have other non-specific actions. Besides interneuronal GJC between dendrites, inter-axonal and inter-glial GJC is also considered important for seizure generation. Interestingly, in most studies of cerebral tissue from animal seizure models and from human patients with epilepsy, there is up-regulation of glial, but not neuronal gap junctional mRNA and protein. Significant changes in the expression and post-translational modification of the astrocytic connexin Cx43, and Panx1 were observed in an *in vitro* Co^++^ seizure model, further supporting a role for glia in seizure-genesis, although the reasons for this remain unclear. Further suggesting an involvement of astrocytic GJC in epilepsy, is the fact that the expression of astrocytic Cx mRNAs (Cxs 30 and 43) is several fold higher than that of neuronal Cx mRNAs (Cxs 36 and 45), and the number of glial cells outnumber neuronal cells in mammalian hippocampal and cortical tissue. Pannexin expression is also increased in both animal and human epileptic tissues. Specific Cx43 mimetic peptides, Gap 27 and SLS, inhibit the docking of astrocytic connexin Cx43 proteins from forming intercellular gap junctions (GJs), diminishing spontaneous seizures. Besides GJs, Cx membrane hemichannels in glia and Panx membrane channels in neurons and glia are also inhibited by traditional gap junctional pharmacological blockers. Although there is no doubt that connexin-based GJs and hemichannels, and pannexin-based membrane channels are related to epilepsy, the specific details of how they are involved and how we can modulate their function for therapeutic purposes remain to be elucidated.

## Introduction

When one thinks of the “connections” between epilepsy and gap junctions (GJs), the usual interpretation is that the GJs form direct intercellular cytoplasmic connections between neurons, promoting the hypersynchronous neuronal activity associated with seizures. However, as this review discusses, that concept is rather naïve and greatly complicated by much new data concerning the potential roles of gap junctional communication (GJC) and membrane Cx hemichannels and pannexin channels. (Giaume et al., 2013). Although there are many other relevant reviews of GJs and epilepsy (Dudek et al., 1998; Carlen et al., 2000; Perez Velazquez and Carlen, 2000; Traub et al., 2004; Nemani and Binder, 2005; Salameh and Dhein, 2005; Jin and Chen, 2011; Carlen, 2012; Steinhauser et al., 2012), the roles of pannexins and Cx hemichannels are mainly ignored.

## Strategic locations of electrotonic communication and seizure activity

It is commonly assumed that the key location for GJC and seizure generation is between neurons, usually between dendrites (Vazquez et al., 2009). For example our group demonstrated GJC between stratum oriens interneurons in the more electrotonically remote distal dendrites based on the relatively low coupling coefficients and the available anatomical evidence (Zhang et al., 2004). However, other locations for GJC critical for seizure generation have now been proposed. Using a double knockout (dKO) of the glial connexins 30 and 43 (Wallraff et al., 2006; Rouach et al., 2008) demonstrated that GJC between astrocytes for the delivery of glucose or lactate to astrocytes was necessary to sustain excitatory synaptic transmission and epileptiform activity. GJC also can spread apoptotic signals (Lin et al., 1998; Andrade-Rozental et al., 2000), a process which could be quite relevant in severe seizure activity (Belousov and Fontes, 2013).

An *in vivo* and *ex-vivo* study of adult rats treated with 4-aminopyridine (4-AP), a K^+^ channel blocker which induces seizures, have shown that dephosphorylation of connexin 43 associated with astrocytic swelling, resulted in reduction of astrocytic gap junction permeability (Zador et al., 2008). These results suggest that, during acute seizures, a prolonged inhibition of intercellular coupling develops in the astrocytic network. Long-lasting (weeks) astrocyte swelling was observed following seizures in the kindling seizure model (Khurgel and Ivy, 1996). It is well-known that glial swelling decreases the cerebral extracellular space (Dietzel and Heinemann, 1986; Sykova, 2004; Badaut et al., 2011). This enhances ephaptic transmission which promotes seizure activity (Jefferys, 1995; Dudek et al., 1998; Shahar et al., 2009). Since astrocytes play an important functional role in extracellular K^+^ and pH homeostasis, pathological brain states that result in K^+^ and pH dysregulation may also cause astrocyte swelling (Florence et al., 2012). The presence of gliotic scars in chronic focal epilepsy patients has led to the suggestion that glia can play an important pathophysiological role in chronic epilepsy (De Lanerolle et al., 2010). The astrocytes in sclerotic hippocampi differ from those in non-sclerotic hippocampi in their membrane physiology and related microvasculature (De Lanerolle et al., 2010). In addition, within these sclerotic hippocampal tissues, there is increased expression of many molecules normally associated with immune and inflammatory functions.

Traub and colleagues have introduced the concept of inter-axonal GJC playing a significant role in seizure-genesis and in the generation of sharp wave ripple complexes (Traub et al., 2002, 2005; Simon et al., 2013). Another underexplored feature of GJC in the CNS is the role of combined electrochemical synapses (Pereda, 2014) well-established in the invertebrate CNS, and recently demonstrated in mossy fiber terminals of the hippocampus by several groups (Hamzei-Sichani et al., 2007; Nagy, 2012; Vivar et al., 2012). What role these synapses play in epilepsy is presently unknown.

## Gap junctions, sharp wave-ripple complexes and seizures

**Figure 1:**
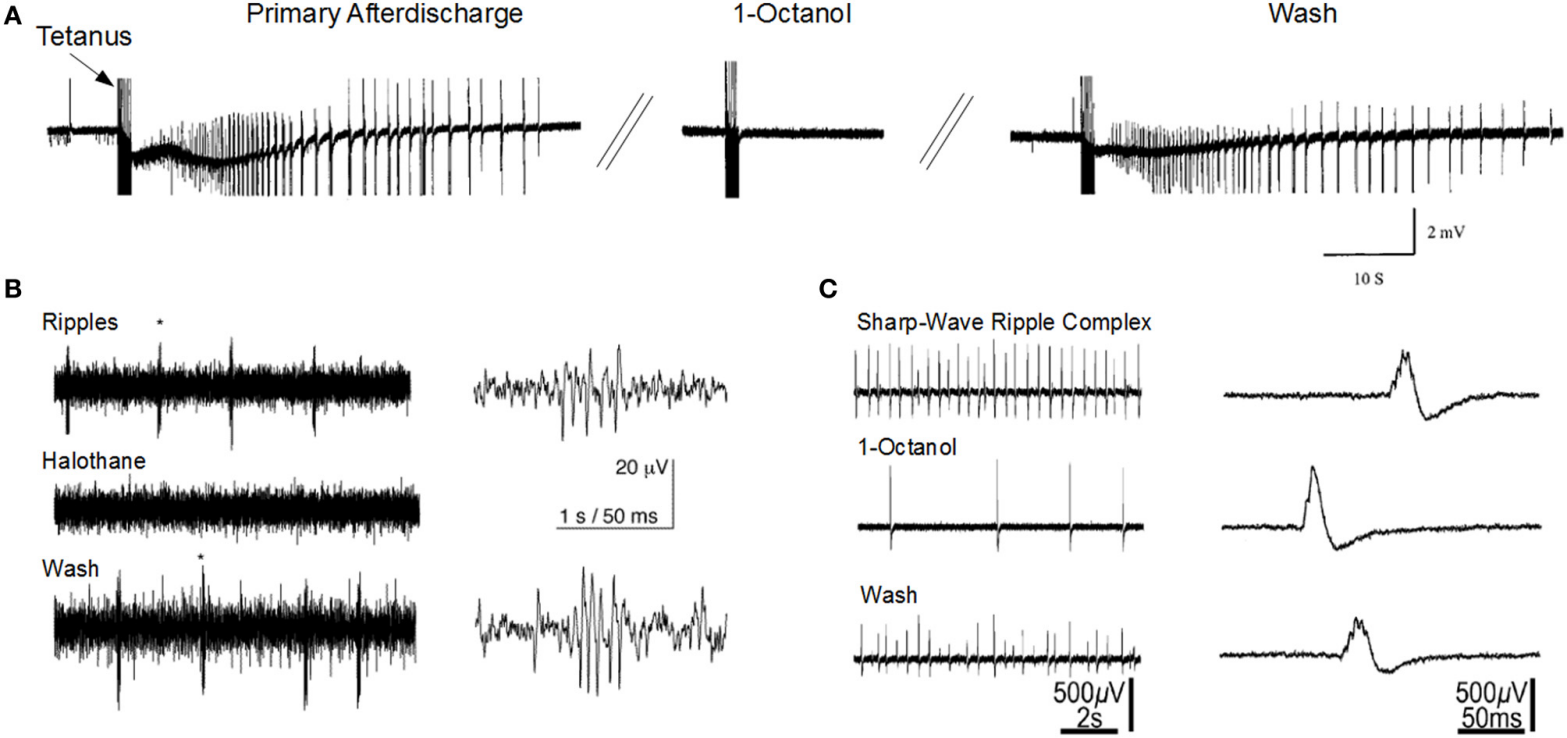
**Gap junction blockers inhibit seizure-like activity and sharp-wave ripple activity in the CA1 hippocampal slice. (A)** 1-Octanol abolishes primary afterdischarge induced after repeated tetanic stimulation for 2 s at 100 Hz (Modified with permission from Jahromi et al., 2002). **(B)** Halothane abolishes high-frequency ripple oscillations that are independent of sharp-wave activity (Modified with permission from Draguhn et al., 1998). **(C)** 1-Octanol reduces sharp-wave ripple activity (Modified with permission from Maier et al., 2003). Asterisks (*) indicate the ripples corresponding to the right traces.

The sharp-wave ripple complex (SPW-ripple) (Figures 1B,C) is highly synchronous physiological activity that is generated in the hippocampus. Emerging evidence suggests that under pathological conditions the neuronal assemblies generating SPW-ripples may also be responsible for generating epileptiform activity (Staba et al., 2004; Khosravani et al., 2005; Behrens et al., 2007; Bragin et al., 2007; Beenhakker and Huguenard, 2009; Liotta et al., 2011; Simeone et al., 2013).

The mechanisms responsible for ripple generation are not fully understood, however a prominent theory suggests that gap junction proteins play a critical role (Figures 1B,C). Traub and colleagues modeled hippocampal ripple activity through axo-axonal electronic coupling of pyramidal neurons (Traub et al., 2002). According to this model, spontaneously generated action potentials in CA1 axons depolarize electronically coupled neurons, resulting in a wave of spike generation that travels along the axonal plexus, and is detected in the LFP as a high frequency oscillation (Traub et al., 1999; Simon et al., 2014). The first experimental evidence implicating a role for GJs in high-frequency ripple oscillations in the hippocampus, came from utilizing multisite single unit recordings in the awake rat (Ylinen et al., 1995). They inhibited ripple oscillations in the CA1 by application of the anesthetic drug halothane, which, along with other effects, also blocks GJs. Subsequently, ripple oscillations were observed in extracellular field recordings in rat hippocampal brain slice preparations (Draguhn et al., 1998; Traub et al., 2002). The ripple oscillations were enhanced following bath application of a calcium-free solution, which enhances GJC and membrane excitability (Perez-Velazquez et al., 1994). Similar to ripples recorded *in vivo*, their occurrence was completely inhibited by pharmacological agents known to block GJs, such as octanol, carbenoxolone, halothane, and low pH.

However, as noted below, gap junction modulators are non-specific, and may mediate their effects through gap junctional-independent mechanisms (Nakahiro et al., 1991; Rouach et al., 2003). Recently, (Behrens et al., 2011) utilized a more specific gap junction blocker, mefloquine, which blocks Cx36, Cx43, and Cx50, and observed no effect on SPW or associated ripples. In Cx36 knockout mice, *in vivo* ripple oscillations were unaltered in terms of power, frequency, or probability of occurrence (Buhl et al., 2003). In contrast, *in vitro* studies on Cx36 knockout mice, demonstrated a reduced frequency of SPW and associated ripples (Pais et al., 2003). However, other *in vitro* studies in Cx36 knockout mice have reported no significant differences in ripple oscillations when chemical synaptic transmission was absent (Hormuzdi et al., 2001) or during application of the glutamate receptor agonist, kainate (Pais et al., 2003). It has to be recognized also that upregulation of other Cxs could significantly alter/blunt the effects of eliminating Cx36. Future studies should investigate the possibility that alternative connexins, connexin hemichannels, or pannexins are involved in the generation of SPW and ripple rythmogenesis.

## Cx hemichannel and PanX channel involvement in seizure activity

GJ channels provide the basis for direct cell-to-cell communication, whereas Cx hemichannels and Panx channels allow the exchange of ions and signaling molecules such as ATP, NAD+, glutamate and other molecules less than 1000 Daltons between the cytoplasm and the extracellular milieu (Willecke et al., 2002). Recent papers have discussed the different properties of Cx hemichannels and Panx channels from the perspective of ATP release (Lohman and Isakson, 2014) and electrophysiological characteristics (Patel et al., 2014). These hemichannels and channels support autocrine and paracrine communication through a process called “gliotransmission,” which involves the uptake and release of metabolites such as glucose and glutathione and also the release of autocrine/paracrine molecules such as ATP, glutamate, NADP+ and adenosine into the extracellular medium (Sohl and Willecke, 2004; Bruzzone et al., 2005; Cherian et al., 2005; Retamal et al., 2007; Lin et al., 2008; Stridh et al., 2008). Studies have shown that the opening of Cx43 hemichannels is promoted by positive transmembrane voltages and reduced concentrations of extracellular divalent cations such as Ca^2+^. High intracellular H^+^, and phosphorylation of Cx43 promotes the closure of the hemichannels (Chang et al., 1996; Bennett et al., 2003; Saez et al., 2005; Verselis and Srinivas, 2008). The release of ATP and glutamate from astrocytic Cx hemichannels induces neuronal death through activation of Panx1 hemichannels (Orellana et al., 2011), a process which may contribute to the increased apoptosis observed in epileptic tissue. Furthermore, astrocytes release ATP in response to raised intracellular Ca^2+^, which subsequently is broken down to adenosine. Adenosine, a neuromodulator, can inhibit presynaptic neurotransmitter release via the activation of P2X receptors (Pascual et al., 2005; Montana et al., 2006; Zhang et al., 2007; Pankratov et al., 2009).

## Effects of GJ channel blockers on seizure activity

The strongest evidence linking GJs and seizures is the seizure-blocking actions of agents that disrupt connexon-based GJ intercellular communication in *in vitro* and *in vivo* epilepsy models (e.g., Figure 1A). Although there are several GJC blockers available which possess anticonvulsive actions, they are not specific and do not usually discriminate between Cx isoforms or cell types (Perez-Velazquez et al., 1994; Ross et al., 2000; Kohling et al., 2001; Rozental et al., 2001; Jahromi et al., 2002; Steinhauser and Seifert, 2002; Szente et al., 2002; Gajda et al., 2003; Samoilova et al., 2003; Bostanci et al., 2006; Gigout et al., 2006; Nilsen et al., 2006; Bostanci and Bagirici, 2007; Medina-Ceja et al., 2008; Giaume and Theis, 2010). Carbenoxolone is one of the drugs widely used a GJ blocker, although one report showed a proconvulsive effect (Voss et al., 2009). Intracellular alkalinization, such as caused by trimethylamine and ammonium chloride, increases GJC, and also enhances epileptiform activity (Perez-Velazquez et al., 1994; Kohling et al., 2001). An antimalarial drug, quinine strongly blocks neuronal GJCs formed by Cx36 and to a lesser degree, Cx45 (Srinivas et al., 2001), but paradoxically enhances *in vitro* neocortical seizure activity caused by low magnesium perfusion, possible by blocking inhibitory interneuronal synchrony (Voss et al., 2009). Compounds of the fenamates family have also been reported to inhibit GJs composed of Cx43 with the following order of efficacy; meclofenamic acid > niflumic acid > flufenamic acid (Harks et al., 2001). Flufenamic acid was also shown to inhibit GJs composed of Cx26 and Cx32, but the selectivity was reported to be low (Srinivas and Spray, 2003). Most of the gap junctional blockers were also shown to affect Cx hemichannels and Panx channels (Giaume and Theis, 2010). Gap junctional blocking agents also affect the conductance of Panx membrane channels (Thompson and Macvicar, 2008; Giaume et al., 2013). Compared to Cx channels, Panx1 channels and Cx hemichannels are even more sensitive to most GJ channel blockers including carbenoxolone (CBX), flufenamic acid, mefloquin (MFQ) (Bruzzone et al., 2005; Iglesias et al., 2008) and Cx mimetic peptides (Dahl, 2007).

Several of the compounds from the fenemate family of blockers block Panx1 hemichannels in the following order: mefloquine > carbenoxolone > flufenamic acid (Iglesias et al., 2009). Studies have also shown that Panx1 hemichannels are 1000- to 10,000-fold more sensitive to mefloquine than Cx43 GJCs (Cruikshank et al., 2004; Iglesias et al., 2008). Although substances such as gadolinium (Gd^3+^) and lanthanum (La^3+^) block hemichannels without inhibiting gap junction or Panx1 channels, these ions are not specific to GJ proteins since they also block other channels such as Ca^2+^ channels (Mlinar and Enyeart, 1993; Liu et al., 2008). Long-chain alcohols are known to block Cx hemichannels but have only a very small effect on Panx channels. In contrast, low concentrations (5–10 μ M) of carbenoxolone block Panx1 channels and only have a minor inhibitory effect on Cx hemichannels (Schalper et al., 2008).

## Effect of mimetic peptides on seizure activity

Connexin mimetic peptides, which correspond to the extracellular loops of connexins, are a class of specific and reversible inhibitors of GJC (Evans and Boitano, 2001; Herve and Dhein, 2010). In rat organotypic cultures, which show spontaneous epileptiform activity, prolonged (>10 h) application of Cx43 mimetic peptides, reduced the spontaneous seizure activity (Samoilova et al., 2008). This long period of incubation was required presumably to block the docking of homologous Cx43 hemichannels, a process which forms GJs, thereby diminishing inter-astrocytic GJC. These data suggest that enhanced inter-astrocytic gap junctional based coupling promotes seizure activity. Under normal physiological conditions, the probability of hemichannels opening is low because of the blocking effects of divalent extracellular ions such as magnesium and calcium (Ebihara, 2003; Ebihara et al., 2003). But when extracellular magnesium and/or calcium levels are reduced, for example, in the case of low Mg^2+^ or low Ca^2+^ seizure models, or the reduced extracellular Ca^2+^, which is seen during seizures in several seizure models (Lux and Heinemann, 1978; Somjen, 2002) the role of hemichannels and the effects of mimetic peptides must also be considered. In this case, higher concentrations of mimetic peptides are presumed to be required in order to block both GJ channels as well as hemichannels (Ebihara, 2003; Ebihara et al., 2003). In a slice culture model wherein added bicuculline caused epileptiform activity, low concentrations of a Cx43 mimetic peptide (amino acid sequence VDCFLSRPTEKT) targeting the extracellular loop two of Cx43, mainly blocked Cx43 hemichannels and prevented seizure-induced neuronal death (Yoon et al., 2010). Higher doses of the peptide, which inhibited GJs in addition to the hemichannels, increased the severity of the seizure-induced lesion. These data suggest that in this model, the neuronal damage from epileptiform neuronal hyperactivity is prevented by Cx43 gap junctional blockade and exacerbated by Cx43 hemichannel opening.

## Gene expression, genetics, and the GJ proteome

Developmental age plays a role in gap junctional expression and seizure susceptibility. It is well-known that immature humans and animal models are more susceptible to undergoing seizures than mature adults (Rakhade and Jensen, 2009). Also expression of glial and neuronal connexins is developmentally regulated (Rozental et al., 2000; Montoro and Yuste, 2004). Within the developing neocortex, significant GJC was measured in four compartments (Sutor and Hagerty, 2005); (1) gap junction-coupled neuroblasts of the ventricular zone and GJs in migrating cells and radial glia, a compartment which disappears with maturity; (2) gap junction-coupled glial cells (astrocytes and oligodendrocytes); (3) gap junction-coupled pyramidal cells (which exists primarily during the first two post-natal weeks in rodents); and (4) gap junction-coupled inhibitory interneurons. It is hypothesized that the increased GJC between pyramidal cells plays a major role in lowering the seizure threshold in immature animals, but the roles played by the age-dependent differential expression of the various gap junctional and pannexin proteins remain unclear. Among the 21 known members of Cxs and three Panxs, it has been determined that there are at least 10 Cxs and 2 Panxs expressed in the brain (Willecke et al., 2002; Baranova et al., 2004). Their expression patterns are not uniform and the distribution of different gap junction subtypes is dependent upon location and developmental maturity (Willecke et al., 2002; Baranova et al., 2004).

Analysis of the mRNA expressions of different Cxs and Panxs in the hippocampal tissues from 15 day mice, showed the highest expression of astrocytic Cxs 43 and 30, followed by Panxs, and the lowest expression was observed in the neuronal Cxs 36, 40, and 45, a few orders of magnitude lower than the pannexins and astrocytic connexons (Mylvaganam et al., 2010). Since the half-lives of most Cxs are short, between 1 and 5 h (Herve et al., 2007), changes in Cx expression can rapidly alter Cx levels in cells (Laird, 2010). Recent studies have demonstrated that several mechanisms are involved in the control of Cx expression. In addition, the GJ proteome which includes complex translational and post-translational mechanisms (e.g., phosphorylation) that can introduce changes in protein conformation, activity, charge, stability and localization, can alter downstream signaling pathways that may contribute to the pathophysiology of several neurological diseases (Laird, 2010; Gehi et al., 2011; Chen et al., 2013). Recently, Cx43 phosphorylation and Panx1 glycosylation were shown to occur following *in vitro* Co2+-induced seizures (Mylvaganam et al., 2010). Although Panx regulation has not been explored to the level studied for Cxs, from the functional point of view, Panxs might also show in seizure models, compensatory, overlapping, or unique physiological roles compared to those of Cxs.

### Altered expression in animal models of epilepsy

Studies have shown changes in the expression of GJ mRNAs and proteins or changes in the post-translational modification form of the Cx43 and Panx protein in epileptic animal models (Table 1). Seizure-induced alterations in the expression of astrocytic Cx43 and Cx30, oligodendrocytic Cx32, and neuronal Cx36 have been measured, although there are some contradictory or inconsistent results. These variations may be due to differences in species, age, animal models, and methods of seizure induction, incubation/treatment time points, duration of seizure activity, and the brain regions examined in each study (Jin and Chen, 2011; Steinhauser et al., 2012). In addition, formation and degradation of GJs play an essential role in regulating the level of intercellular communication. With reported half-lives of Cxs being 1–5 h in tissues, the regulation of gap junction assembly and turnover is likely to be critical in the control of GJC. It has also become increasingly clear that Cxs have profound effects on gene expression (Kardami et al., 2007) and the presence of a Cx subtype can also influence the channel formation of other Cx subtypes (Chang et al., 1996).

**Table 1:** **Connexin and pannexin expression changes associated with experimentally-induced seizures in different rodent models**.

**Gene**	**Brain region**	**Seizure model and age**	**Expression change**		**References**
			**mRNA**	**Protein**	
Cx43	Hippocampus	*In vitro* Co^2+^-induced seizure in mouse model PD (post-natal days) 15	2-fold Increase	Increased non-phosphorylated form	Mylvaganam et al., 2010
		Li^+^-pilocarpine induced SE model–PD 30–45 SD rats		Increased in CA1, CA3 and the dentate gyrus	Su and Tong, 2010
		Kainic acid (KA) model using PD 1–14 and adult wistar rats	Decreased in CA3-CA4 pyramidal layers and increased in other regions		Condorelli et al., 2003
			Slight decrease after 4 weeks	Same as mRNA	Sohl et al., 2000
		Bicuculline-Hippocampal organotypic slice cultures from PD 7 wistar rats	Increased	Increased	Samoilova et al., 2003
		Lipopolysaccharide (LPS) induced adult rat model		Decreased	Sayyah et al., 2012
	Amygdala and cerebral cortex	*In vivo*-tetanus toxin induced seizure model using adult rats	Decreased		Elisevich et al., 1998
	Primary focus and mirror focus	*In-vivo* 4-Amino pyridine(4-AP) model (4-AP) using PD 40–50 wistar rats	Increased	Increased	Szente et al., 2002
	Neocortex and hippocampus	Mouse model of tuberous sclerosis complex using PD 14–35 Tsc1^GFAP^CKO mice		Decreased	Xu et al., 2009
Cx30	Cortex, thalamus and amygdaloid nucleus	Kainaic acid (KA)-induced epilepsy in P1, PD7 and 14, and adult wistar rats	Increased within 6 h and decreased after 12 and 24 h	Increased within 6 h	Condorelli et al., 2003
	Hippocampus	KA induced epilepsy in 7–8 weeks old SD rat model		Slightly reduced	Takahashi et al., 2010
				Unchanged	Sohl et al., 2000
			Increased from 6 to 24 h in CA3-CA4 pyramidal layers	Decreased within 12–24 h	Condorelli et al., 2002
Cx 32	Hippocampus	Bicuculline—treated organotypic slice cultures using 7 days old Wistar rats	Increased	Increased	Li et al., 2001; Samoilova et al., 2003
		*In vivo* kindling model using adult rats	Decreased	No change	Sayyah et al., 2012
Cx 36	Amygdala	Adult Wistar Kindling rat model	Increased during focal seizures, then back to basal levels after onset of generalized seizures	Increased during focal seizures, then back to basal levels after onset of generalized seizures	Beheshti et al., 2010
	Hippocampus	Kindling model in PD 26–33 CD rats	Decreased	Decreased	Sohl et al., 2000
Panx1	Hippocampus	Co^++^ mouse model using PD 15 mice	1.5-fold Increase	Increased glycosylated ~48 kDa	Mylvaganam et al., 2010
				Decreased glycosylated ~46 kDa	
				Increased native form ~43 kDa	
Panx2	Hippocampus	Co^++^ mouse model using PD 15 mice	1.4-fold Increase	No change	Mylvaganam et al., 2010

Among the many Cxs present in the brain, studies have reported alterations only in Cx30, 32, 36, and 43 (Table 1). Interestingly, recent studies using dKO mice (Magnotti et al., 2011) observed that animals lacking oligodendrocytic Cx32 and astrocytic Cx43 displayed seizures, motor impairment, and early mortality. Although myelin ultrastructure was not affected, abrupt formation of vacuolation in the white matter and loss of astrocytes was observed in these animals. These observations indicate an unexpected role for specific astrocytic/oligodendrocytic connexins in the survival of astrocytes. When compared to previous studies using Cx43-Cx30 dKO mice, the loss of astrocytic Cx43 and Cx30 (Lutz et al., 2009) was less deleterious than the loss of Cx43 and Cx32. This could mean that Cx32 and Cx43 together mediate signaling events that promote astrocyte survival. However, it is difficult to interpret these results without understanding the nature of the signals and mechanisms underlying the pathology of astrocytes in Cx32-Cx43 dKOs. The first report on seizure-associated Panx expression alterations in the mouse hippocampus, showed a 1.5 increase in Panx1 mRNA and a 1.4-fold increase in Panx2 mRNA (Mylvaganam et al., 2010). Also, a 2-fold increase in Cx43 mRNA and protein were observed in the same study. In addition, Panxs 1 and 2 and glial Cx mRNAs became highly correlated following the seizure activity. We suggested that this marked cross-correlation could be the basis of a transcriptomic network of coordinated gene expression, related to seizure induction or seizure activity (Mylvaganam et al., 2010). Here the highly correlated transcript abundance might also imply that the levels of expression are controlled relative to one another to provide proper functionality of the oligomeric proteins (Mylvaganam et al., 2010). The same study evaluated post-translational modifications and showed a significant increase in glycosylated Panx1 expression after seizures, leading to the notion that involvement of Panx1 hemichannels may contribute to seizures in this model (Mylvaganam et al., 2010). Previous studies have shown that the functional state and cellular distribution of mouse Panxs are regulated by their glycosylation status and interactions among Panx family members (Penuela et al., 2009). Glycosylation sites are located on the extracellular loop and high levels of glycosylated Panx proteins prevented interactions between pannexins (Boassa et al., 2007; Penuela et al., 2007), therefore supporting the formation of membrane channels. Unlike connexins, which are not glycosylated, Panx1 is glycosylated in the second extracellular loop (Boassa et al., 2007; Penuela et al., 2007). This modification adds considerable bulk to the extracellular domain of the protein. Insertion of glycosylation sites into the extracellular loop domains of connexins blocked formation of intercellular junctional channels upon glycosylation (Dahl et al., 1994). Panx1 channel properties are similar to Cx hemichannels. They are activated by depolarization, mechanical stress, raised extracellular potassium, and P2 receptor activation (Scemes et al., 2009; Santiago et al., 2011). Panxs mediate ATP release from astrocytes and neurons (Bao et al., 2004; Iglesias et al., 2008, 2009) suggesting that these channels may contribute to seizures by raising extracellular ATP and arachidonic acid (Thompson et al., 2008; Iglesias et al., 2009; Macvicar and Thompson, 2010). NMDA receptor activation opens Panx channels promoting epileptiform activity (Thompson et al., 2008). Blockade or deletion of Panx1 channels diminished ATP release and seizure activity. However, Kim and Kang showed that P2X7R-Panx1 complex may play an important role as a negative modulator of M1 receptor-mediated seizure activity *in vivo*, since they showed pilocarpine-induced seizures in mice were enhanced following administration of P2X7R antagonists or by gene silencing of P2X7R or Panx1 in WT in a process mediated by PKC via intracellular Ca2+ release (Kim and Kang, 2011).

### Altered GJ expressions in human epilepsy

Gap junction expression studies in human epileptic tissue have demonstrated no change (Elisevich et al., 1997) or elevated (Naus et al., 1991; Collignon et al., 2006) levels of Cx mRNA and protein (Table 2). In addition, altered expression of several membrane channels, receptors, and transporters in astroglial membranes have been found in tissue from epileptic human brain. Although the significance of these alterations is poorly understood, modified astroglial functioning might have an important role in the generation and spread of seizure activity.

**Table 2:** **Gap junctional expression changes associated with human epilepsy**.

**Gene**	**Epileptic condition**	**Brain region**	**Expression change**		**References**
			**mRNA**	**Protein**	
Cx32	Temporal lobe epilepsy (TLE)	Neocortex	Increased		Naus et al., 1991; Jin and Chen, 2011
		Hippocampus	Decreased		Collignon et al., 2006
Cx36	TLE	Hippocampus	Unchanged		
Cx43	Intractable seizure			Increased	Naus et al., 1991
	Complex partial seizure disorder		No significant change		Elisevich et al., 1997
	TLE	Hippocampus Cortex	Increased		Jin and Chen, 2011
	Epilepsy associated brain tumors	Perilesional Cortex		Low-grade gliomas showed Increased expression and different isoforms (like controls) but most high-grade gliomas had only one isoform (non-phosphorylated)	Aronica et al., 2001
	Generalized seizure in the progression of mesial temporal lobe epilepsy	Hippocampus	Increased		Fonseca et al., 2002
Panx1	TLE	Cortex		Increased	Jiang et al., 2013
Panx2				No change	
Panx1+Panx2				Only in layers II and III in the control but present in all layers in TLE patients	

Whether these changes in gene expressions have any direct effect on seizure generation is unclear. These changes might be pro-epileptic responses contributing to the pathogenesis of the disease, or adaptive responses to cope with the pathologic condition. Confounding the interpretation of human data is the fact that epilepsy is not a single condition, but a large group of highly heterogeneous disorders, which have in common an abnormally increased predisposition to seizures (Fisher et al., 2005; Steinhauser et al., 2012). It is difficult to conduct proper GJ expression studies in humans, since tissues obtained from patients are usually at different stages of the condition and seizure duration. Furthermore, type of epilepsy, patient age, duration of seizure and antiepileptic treatment might also alter connexin expression. “Controls” used in most studies are from tumor or autopsy specimens. Hence apparent changes in connexin levels could be caused by altered expression in “control” tissues (Nemani and Binder, 2005). Also, changes in mRNA or protein levels do not necessarily translate into changes in functional coupling. Therefore, functional assays are required for the reliable investigation of the role of GJs in human epilepsy. Neurosurgical specimens from patients presenting with TLE are often accompanied by massive reactive gliosis (Hinterkeuser et al., 2000; De Lanerolle and Lee, 2005; Kim and Kang, 2011) and activated astrocytes (Hinterkeuser et al., 2000; Seifert et al., 2006). Also in human tissue and in animal models of mesial temporal lobe epilepsy there are alterations in expression, subcellular localization, and function of astroglial GJs which might impair K+ buffering and other homeostatic network functions (Heinemann et al., 2000; Kivi et al., 2000). Evidence also suggests that in TLE patients, changes in glia can alter the delivery of energetic substances to neurons and consequently lead to a short term functional alterations in neurons. Dysfunction of astrocytic K+ homeostasis and adenosine function has been shown to play a major role in epileptogenesis (Dityatev and Rusakov, 2011; Allen et al., 2012; Risher and Eroglu, 2012; Wang et al., 2012; Boison et al., 2013). In a recent study, quantitation of Panx1 and Panx2 expression in surgically removed human brain tissue of epileptic temporal lobes showed that Panx1 and Panx2 proteins were expressed in all layers of the epileptic cortex, but predominantly restricted to layers II and III of the control group (Jiang et al., 2013). Overall, Panx1 protein expression was significantly higher in the temporal lobe cortex of patients with TLE compared to controls. No significant differences were identified in Panx2 expression levels. This is the first study to show that Panx channels may also be involved in the pathogenesis of human epilepsy. These expression changes could be a cause or a response to the epileptic neuronal hyperactivity or an effect of chronic epileptic damage. Further studies are required.

### Cx genetic mutations associated with epilepsy

At present, there are at least 10 distinct diseases known to be associated with gene mutations in connexins, some of which are associated with epilepsy. Connexin-linked diseases caused by gene mutations or altered connexin expression, protein assembly or localization, will ultimately impact the rest of the proteome, which can influence the manifestation of the pathology (Laird, 2010). In humans, the vast majority of epileptic cases are of idiopathic origin, thus a deeper understanding of the cellular and molecular mechanisms will lead to new insights into brain structure and function as well as improved therapies. Recent studies have indicated that Cx36 is a likely gene linked to Juvenile myoclonic epilepsy (JME). A case control study performed on a sample of 29 JME patients with a mutationin15q14 loci, 140 randomly selected JME patients, and 123 controls, demonstrated a significant association between JME and Cx36 gene (Mas et al., 2004; Hempelmann et al., 2006).

Mutations of the tumor suppressor genes *TSC1* and *TSC2* are found in tuberous sclerosis, and epilepsy is one of its major manifestations. Deleting *TSC1* in astrocytes caused seizures attributed to diminished astrocytic GJ intercellular communication and impaired potassium buffering (Xu et al., 2009). Patients with oculodentodigital dysplasia (ODDD), a rare genetic disease which is caused by mutations in the gene encoding Cx43, develop seizures in addition to other neurological symptoms (Loddenkemper et al., 2002). In cell culture, some of the ODDD-associated mutations of Cx43 cause loss of GJ coupling, but increased hemichannel activity (Dobrowolski et al., 2007).

## Conclusions and questions

In brief, the roles of GJs, connexins, and pannexins in epilepsy remain unclear. The lack of specificity of pharmacological agents now used to block or enhance GJC is a challenge if one wants to understand the role of a particular type of connexin-based GJ, and if wants to develop a specific therapeutic tool. The fact that most of these agents affect the Cx hemichannels and the Panx membrane channels further confuses the interpretation of experimental data. The ongoing development of peptides targeting specific Cxs and Panxs will permit a more precise dissection of the functions of the different Cx and Panx species. Although the gap junctional blocking drugs are almost always anticonvulsant, there is not as of yet an anticonvulsant drug on the market that is reputed to have gap junctional blocking properties, although both acetazolamide and topiramate inhibit carbonic anhydrase activity which should cause an intracellular acidosis thereby blocking GJC. Another problem is the overwhelming evidence that tissue from animal models and human epileptics show increases in Cxs expression in glia but not in neurons. This begs the question as to what are the roles of glia in seizure generation. If applying a gap junctional blocking agent to epileptic tissue is blocking interglial GJC, then what is the underlying anticonvulsant mechanism? Or could these agents have their major anticonvulsant action by blocking the conductance of Cx hemichannels and/or Panx channels? The scientific community is just now starting to consider the possible role of these membrane channels in the pathophysiology of epilepsy. In summary, although there is no doubt that GJs, connexins and pannexins are intimately related to epilepsy and seizure generation, the specific details of exactly how they are involved and how we can modulate their function for therapeutic purposes remain to be elucidated.

### Conflict of interest statement

The authors declare that the research was conducted in the absence of any commercial or financial relationships that could be construed as a potential conflict of interest.
